# Bridging Synaptic and Epigenetic Maintenance Mechanisms of the Engram

**DOI:** 10.3389/fnmol.2018.00369

**Published:** 2018-10-05

**Authors:** Madeleine Kyrke-Smith, Joanna M. Williams

**Affiliations:** ^1^Department of Anatomy, The Brain Health Research Centre, Brain Research New Zealand – Rangahau Roro Aotearoa, University of Otago, Dunedin, New Zealand; ^2^Department of Psychology, The Brain Health Research Centre, Brain Research New Zealand – Rangahau Roro Aotearoa, University of Otago, Dunedin, New Zealand; ^3^Department of Neurobiology and Anatomy, University of Utah, Salt Lake City, UT, United States

**Keywords:** epigenetics, learning, LTP, memory, plasticity

## Abstract

How memories are maintained, and how memories are lost during aging or disease, are intensely investigated issues. Arguably, the reigning theory is that synaptic modifications allow for the formation of engrams during learning, and sustaining engrams sustains memory. Activity-regulated gene expression profiles have been shown to be critical to these processes, and their control by the epigenome has begun to be investigated in earnest. Here, we propose a novel theory as to how engrams are sustained. We propose that many of the genes that are currently believed to underlie long-term memory are actually part of a “plasticity transcriptome” that underpins structural and functional modifications to neuronal connectivity during the hours to days following learning. Further, we hypothesize that a “maintenance transcriptome” is subsequently induced that includes epigenetic negative regulators of gene expression, particularly histone deacetylases. The maintenance transcriptome negatively regulates the plasticity transcriptome, and thus the plastic capability of a neuron, after learning. In this way, the maintenance transcriptome would act as a metaplasticity mechanism that raises the threshold for change in neurons within an engram, helping to ensure the connectivity is stabilized and memory is maintained.

## Introduction

Networks of strongly connected neurons form the physical trace of declarative, non-declarative and emotional memories as well as habits, sensory associations and motor function (Takeuchi et al., 2013; Josselyn et al., 2015; Eichenbaum, 2016; Poo et al., 2016; Tonegawa et al., 2018). This concept of a physical trace of memory, consisting of networks of cells that have undergone synaptic strengthening and thus preferentially fire together, was postulated by Donald Hebb (Hebb, 1949). Since then, there have been competing arguments throughout the literature about the name given to the physical trace. Here, we refer to the cells activated and involved in a memory, either through experience or through artificial enhancement, as an engram.

The ensemble of neurons involved in an engram is critical to the uniqueness of each memory. Artificially activating an established engram, at the same time as learning something new, can associate the two engrams and thus create a “false memory” without the two events ever being associated in “real life” (Liu et al., 2012; Ramirez et al., 2013, 2015). Further, associating a weak learning experience with a strong learning experience causes the respective engrams to overlap, and the retrieval of those associated events then requires the activation of the two overlapping engram (Cai et al., 2016; Nomoto et al., 2016; Abdou et al., 2018). Moreover, disrupting the connectivity of an established engram can erase a memory (Nabavi et al., 2014; Hayashi-Takagi et al., 2015; Roy et al., 2016) and re-connecting or activating the same group neurons can restore it (Nabavi et al., 2014; Roy et al., 2016). Indeed, *in vivo* visualization of specific synapses modified by a learning event has recently been achieved (Choi et al., 2018). The engram is therefore dependent upon mechanisms which can selectively enhance and refine the synaptic connectivity of neurons. Numerous mechanisms have been identified that can modify synaptic connectivity and are collectively referred to as *synaptic plasticity* (Citri and Malenka, 2008). However, cell to cell communication is not just dependent upon synaptic transmission. The response of a cell to synaptic activation also depends upon the subsequent conductance of current through the dendritic tree, the depolarization and repolarization at the soma and the conductance of action potentials along the axon (Hausser et al., 2000; Beck and Yaari, 2008; Larkum and Nevian, 2008; Kastellakis et al., 2015). These processes reflect the intrinsic excitability of a neuron (Zhang and Linden, 2003). Thus, engram formation and maintenance is also likely to be critically dependent on modifications to excitability, so called *intrinsic plasticity* (Zhang and Linden, 2003; Mozzachiodi and Byrne, 2010). Recently, Lisman and colleagues argued strongly for the incorporation of both synaptic and intrinsic plasticity mechanisms into our understanding of memory formation (Lisman et al., 2018). Together, this suggests that there is a central, coordinated response to a learning event, leading to adaptations throughout a given neuron. This central response necessitates a central mechanism, or regulator, to determine successful engram formation and maintenance.

Long-term potentiation (LTP) is an activity dependent and input specific synaptic plasticity mechanism which manifests as enhanced transmission between pre- and post-synaptic regions (Bliss and Gardner Medwin, 1973; Bliss and Lømo, 1973; Bliss and Collingridge, 1993). There is strong support for LTP as the candidate mechanism which incorporates and maintains the specific connectivity of an engram (Bliss and Collingridge, 1993; Abraham and Williams, 2003; Takeuchi et al., 2013; Poo et al., 2016; Choi et al., 2018). The most striking evidence comes from experiments showing that LTP occurs at the same time as learning and that learning occludes further electrically induced LTP (Whitlock et al., 2006; Rioult-Pedotti et al., 2007). Additionally, abolishing the potentiation of the synapses involved in a previously established engram, and then restoring the potentiation using an optogenetic stimulation protocol which has been shown to induce LTP, will erase and then restore the memory, respectively (Nabavi et al., 2014).

Much like learning itself, the induction of LTP also leads to the induction of an array of other synaptic and non-synaptic plasticity mechanisms. For example, less active or inactive synapses surrounding potentiated synapses can be weakened after LTP induction or learning, thereby enhancing the salience of potentiated synapses or removing unwanted inputs (Lynch et al., 1977; Abraham and Goddard, 1983; Caroni et al., 2014; Nakayama et al., 2015). This can be achieved either by long-term depression (LTD) (Lynch et al., 1977; Abraham and Goddard, 1983) or depotentiation of synapses (Caroni et al., 2014; Nakayama et al., 2015). Further, a cell’s intrinsic excitability is also regulated by LTP induction (Andersen et al., 1980; Cai et al., 2016). Thus, LTP, together with the weakening of other synapses and modifications to intrinsic excitability, culminates in changes in the transmission of information within and between neurons after the stimulation protocols used to induce LTP. This evidence supports the notion of a central, coordinated response to plasticity induction. Thus, when considering how LTP or memories are maintained over the long-term it is important to incorporate the cell-wide molecular, anatomical and functional changes throughout the neuron that culminate in the given measured response, be that synaptic strength, cellular activity or memory recall, rather than each individual component alone. Together, this leads to the question; what might be the master regulator of cell-wide plasticity?

It is well accepted that the maintenance of altered synaptic strength, as well as the maintenance of memory, is critically dependent upon changes in gene expression (Goelet et al., 1986; Nguyen et al., 1994). Occurring centrally within the cell, regulated gene expression is a compelling contender as the master regulator of the incorporation of a given neuron into an engram. Seemingly rapid (<1 h post stimulation) changes in the expression of some genes may directly underpin the alterations to synaptic strength by changing the molecular anatomy, and thus function, of the synapses in question (Lyford et al., 1995; Vician et al., 1995; Brakeman et al., 1997; Kato et al., 1997, 1998; Beilharz et al., 1998; Yamagata et al., 1999; Ryan et al., 2011). Simultaneously, substantial changes in the expression of immediate early gene transcription factors (TFs) are concomitant with LTP induction (Cole et al., 1989; Wisden et al., 1990; Jones et al., 2001; Abraham and Williams, 2003, 2008; Ryan et al., 2011). Activation of these TFs have been ascribed to underpinning persistent alterations in synaptic anatomy and strength, by the replenishment of synaptic molecules. However, cellular activity (Dudek and Fields, 2002; Tyssowski et al., 2018), muscarinic or β-adrenergic receptor activation (Frey et al., 2001) or dopamine activity (Sajikumar and Frey, 2004) all induce similar patterns of gene expression to that which is induced by stimulation protocols that lead to LTP. This suggests that some of the early, transient changes in gene expression that are observed following LTP induction are related to activity, rather than LTP alone. Further, due to the temporal and spatial spread of gene expression after LTP induction and learning, the early gene response may not be specific to the maintenance of an engram over the long-term.

### Statement of Hypothesis

Here, we propose that the genes that are currently associated with LTP and long-term memory (LTM) are part of a “plasticity transcriptome,” which reflects a transient “up-state” in neuronal activity at the time of learning or upon the induction of synaptic plasticity. The plasticity transcriptome functions to orchestrate dramatic, widespread changes to the structure and function of a neuron, underpinning altered synaptic plasticity and intrinsic excitability. However, in order to allow both consolidation of the engram and ongoing plasticity within a network of neurons a “maintenance transcriptome” develops over hours to days after LTP induction or learning and creates a new *metaplastic state*. Metaplasticity refers to a shift in the state of a cell that alters the ease or type of plasticity that can be induced by a given stimuli (Abraham and Bear, 1996; Abraham, 2008). We hypothesize that epigenetic mechanisms, in particular histone deacetylation, are central to the maintenance transcriptome and act as the *master negative regulators* of plasticity by controlling the expression of the plasticity transcriptome, i.e., acting as a metaplastic regulator of gene expression. Accordingly, the ability to modify the structure and function of neurons within a given engram would be attenuated. This epigenetic metaplastic rise in the threshold for change would preferentially maintain the structure of the engram whilst allowing for plasticity within the network, should the new threshold for plasticity be met. This hypothesis is based on the key observations that while gene expression is required for LTM, it is not exclusively related to synaptic enhancement, that canonical gene expression is not sufficient to maintain LTM and that epigenetic mechanisms are activated late after the induction of LTP and regulate plasticity related gene expression.

## Long-Term Potentiation

Long-term potentiation of specific synapses can be achieved in a myriad of ways, including by real learning experiences (Whitlock et al., 2006), or by *in vivo* (Bliss and Gardner Medwin, 1973; Bliss and Lømo, 1973; Douglas and Goddard, 1975; Abraham et al., 2002) or *in vitro* electrical stimulation (Schwartzkroin and Wester, 1975; Alger and Teyler, 1976; Andersen et al., 1980). Infusion of neurotropic factors such as Brain Derived Neurotropic Factor (BDNF) either directly into the brain (Akaneya et al., 1997) or by application to neuronal slice preparations (Kang and Schuman, 1995) can also enhance synaptic transmission, as can a variety of chemical agents modulating glutamatergic and other receptors (Reymann et al., 1986; Thibault et al., 1989; Aniksztejn and Ben-Ari, 1991; Frey et al., 1993; Lu et al., 2001; Fujii et al., 2002; Otmakhov et al., 2004). Therefore, our understanding of the molecular underpinnings of LTP are derived from diverse study protocols, each likely modeling only some aspects of the ensemble of events which come together as a net enhancement in synaptic strength over the long term.

When induced *in vivo* LTP, like memories, can last for months (Abraham et al., 2002; Abraham, 2003). Electrically-induced LTP is often separated into 2–3 categories. Early LTP (E-LTP or LTP1), lasts only minutes (*in vitro*) to hours (*in vivo*) and depends upon post-translational modifications to proteins present at activated synapses (Shirke and Malinow, 1997; Benke et al., 1998). These modifications lead to enhanced currents through receptors, increased numbers of receptors in the postsynaptic density (PSD) and enhanced presynaptic transmitter release (Dolphin et al., 1982; Shirke and Malinow, 1997; Benke et al., 1998). By contrast, late LTP (L-LTP), which arguably is most reliably studied *in vivo* in the dentate gyrus (DG), lasts for much longer. L-LTP can be divided into an intermediate form, LTP2, which can last for days *in vivo*, and LTP3 which can last weeks to months *in vivo* (Abraham et al., 2002; Abraham, 2003; Abraham and Williams, 2003) and is believed to underlie LTM.

Both LTP2 and LTP3 depend upon the synthesis of new plasticity-related proteins (PRPs) (Frey and Morris, 1997) thought to further modify the structure of the synapse (Sacktor et al., 1993; Frey and Morris, 1997; Raymond et al., 2000). Unlike LTP2, LTP3 critically depends upon rapid changes in gene expression. A compelling body of literature shows that a subset of these genes function to expand the range of newly synthesized PRPs available to modify synapses or replenish molecules directly involved in enhancement of synaptic transmission (Abraham and Williams, 2003; Alberini and Kandel, 2015; Sweatt, 2016). These include genes such as *Homer* (Brakeman et al., 1997; Kato et al., 1997, 1998), *Arc* (Link et al., 1995; Lyford et al., 1995), *Arcadlin* (Yamagata et al., 1999), *RB-3* (Beilharz et al., 1998), *Syt4* (Vician et al., 1995; Ryan et al., 2011) and *Nrxn3, Adrb1, Grm6, Chrm4, Chrna4, Grin2D, Gad2* (Ryan et al., 2011). Regulation of the expression of these genes may underpin the structural plasticity at the synapse proposed by Caroni et al. (2014) where synaptic growth, occurring 1–2 h after learning, is followed by strengthening of specific synapses over 12–18 h and the elimination of spines over the following 1–2 days (Caroni et al., 2014). We hypothesize that these genes comprise the “plasticity transcriptome.” Furthermore, transcription factors such as *zif/268/Egr1, erg2, egr3 c-jun and jun-b* (Cole et al., 1989), and *c-fos* (Cole et al., 1989; Dragunow et al., 1989) are upregulated simultaneously with the so-called effector genes described above and correspondingly stimulate subsequent waves of gene expression (Nguyen et al., 1994; Dudai, 1996; Abraham, 2003; Abraham and Williams, 2003; Medina et al., 2008; Neves et al., 2008; Josselyn et al., 2015; Tyssowski et al., 2018). The contribution of this mass upregulation of TFs to the maintenance of long-lasting engrams is as yet not adequately resolved.

Interestingly, though the nomenclature of LTP suggests that L-LTP may be an extension of E-LTP, or that LTP1, 2 and 3 may be a continuum, this has not been explicitly proven and indeed, evidence suggests that they may be distinct. The processes necessary for L-LTP, namely protein synthesis and gene expression, are initiated at the same time as LTP is induced, rather than subsequently (Cole et al., 1989; Abraham et al., 1991; Bito et al., 1996; Raymond and Redman, 2006; Benito and Barco, 2015). Further, activation of distinct signaling pathways are needed to induce each type of LTP (Raymond and Redman, 2006). These pathways are not necessarily dependent upon each other but may, or may not, be activated at the same time depending on the induction paradigm (Raymond and Redman, 2006). In particular, stimulation which leads to the induction of LTP1 causes a rise in Ca^2+^ within dendritic spines via N-methyl-D-aspartate receptor (NMDAR) and ryanodine receptor activity (Raymond and Redman, 2006). LTP2 also appears to also be dependent upon NMDAR activation at synapses, but additionally depends upon metabotropic glutamate receptor (mGluR) activation, leading to inositol 1,4,5-trisphosphate receptor (IP_3_R) activated Ca^2+^ release within dendrites to drive local protein synthesis (Raymond et al., 2000; Raymond and Redman, 2006). Finally, LTP3 has a significant NMDAR independent component which is instead dependent upon Ca^2+^ rise through L-type voltage-gated calcium channels (L-type VGCC) at the cell body (Raymond and Redman, 2006). Thus, for this paper we take the perspective that LTP3 may well be an independent form of LTP and, as such, must be investigated independently of LTP1 and LTP2. Transcription-dependent LTP is referred to as L-LTP or LTP3 depending upon the classification made within the paper referenced.

### Gene Expression Initiated Upon L-LTP Induction and Learning Is Not Exclusively Related to the Enhancement of Synapses

**FIGURE 1:**
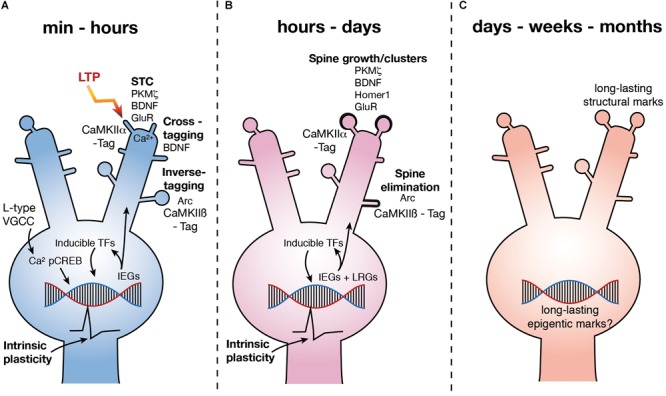
Molecular mechanisms underlying LTP3. **(A)** Upon LTP induction, Ca^2+^ influx via L type VGCCs leads to the phosphorylation of CREB. pCREB stimulates the expression of a number of IEG’s leading to the production of PRPs, some of which are involved in synaptic plasticity via STC (potentiation at CaMKIIα tagged synapses), inverse tagging (depotentiation or depression at CaMKIIβ tagged synapses) and cross-tagging (synaptic depression) mechanisms. Other IEG’s are inducible TFs which stimulate subsequent waves of gene expression. Further, activation of CREB is also known to increase excitability (depicted as action potential waveform). **(B)** Over hours to days, subsequent waves of gene expression are involved in spine growth as well as spine elimination. Excitability remains high for at least 5 h (depicted as action potential waveform). **(C)** LTP persists for months, yet how changes in gene expression are regulated and how this regulates functional processes of the neuron over the long term is currently unknown. GluR – subunits of NMDAR and AMPAR.

Upon L-LTP induction, the rapid gene expression response seemingly enables the sustained potentiation of any synapse that is activated to a sufficient degree. Somewhat surprisingly, this degree of activity does not have to be to the extent that would induce L-LTP on its own (Frey and Morris, 1997). The mechanism for this, termed synaptic tag and capture (STC), requires only a “tag” to be set at activated synapses which “captures” newly synthesized PRPs (Frey and Morris, 1997; Redondo and Morris, 2011; **Figure 1**). This tagging phenomenon has also been identified in relation to memory, called instead behavioral tagging, where weak memories can become long-term memories when associated with a strong learning experience which would elicit this central gene response (Bear, 1997; Ballarini et al., 2009; Lesburguères et al., 2011; Redondo and Morris, 2011; Dunsmoor et al., 2015; Nomoto et al., 2016). In addition to the STC mechanisms, an “inverse tag” (Okuno et al., 2012) mechanism and a “cross-tagging” (Sajikumar and Frey, 2004; Sajikumar and Korte, 2011) mechanism have also been identified which allows for the depotentiation or depression of non-potentiated synapses (**Figure 1**). This range of “tags” can be set after L-LTP induction, increasing the diversity of mechanisms in which newly synthesized proteins may be involved (Frey and Frey, 2008; Okuno et al., 2012). Indeed, newly synthesized proteins induced by L-LTP induction can allow for the induction of L-LTD after stimulation that would normally only induce E-LTD (Sajikumar and Frey, 2004; Sajikumar et al., 2007), further corroborating the idea that the gene expression and protein synthesis elicited by L-LTP induction is generalized to many plasticity mechanisms (Kelleher et al., 2004; Sajikumar and Frey, 2004; Sajikumar et al., 2007).

Some PRPs related to L-LTP and L-LTD have been identified (**Figure 1**). The PKC isoform PKMζ has been proposed as one of the key PRP’s involved in STC and has been shown to be essential to the maintenance of L-LTP and LTM (Sajikumar et al., 2005; Sajikumar and Korte, 2011; Tsokas et al., 2016) via an interaction with a CaMKII dependent tag at potentiated synapses (Sajikumar et al., 2007; **Figure 1**). BDNF is also associated with structural changes at potentiated synapses via STC (Barco et al., 2005; Sajikumar and Korte, 2011), as well as via activation of the trkB receptor (Korte et al., 1998) specifically at the synapse which has been potentiated (Harward et al., 2016). However, BDNF also appears to be involved in a cross-tagging mechanism which targets PRPs to synapses at which LTD has been induced (Sajikumar and Korte, 2011; **Figure 1**).

The role of *Arc* in learning and LTP has been thoroughly reviewed (Shepherd and Bear, 2011; Minatohara et al., 2015). Expression of the IEG *Arc* increases rapidly after L-LTP induction (Link et al., 1995; Lyford et al., 1995; Steward et al., 1998; Steward and Worley, 2001; Messaoudi et al., 2007; Ryan et al., 2011; Yilmaz-Rastoder et al., 2011) and plays a role in the structural rearrangement of synapses, in particular the endocytosis of α-amino-3-hydroxy-5-methyl-4-isoxazolepropionic acid (AMPA) receptors (Chowdhury et al., 2006). Inhibition of *Arc* expression 2 h after LTP induction causes LTP to rapidly decay to baseline (Messaoudi et al., 2007) and inhibition of *Arc* prior to LTP induction can restricts its persistence (Guzowski et al., 2000). Further, in mice in which the *Arc* gene has been knocked out (ArcKO) the magnitude of LTP induced in enhanced, but LTP cannot persist and the animals have impaired LTM (Plath et al., 2006). However, it is not clear if Arc is directly involved in the potentiation of synapses as recent work has shown that by interacting with an inverse tag set at inactive synapses by calcium/calmodulin-dependent protein kinase II β (CaMKIIβ), Arc removes AMPA receptors containing GluA1 subunits, thereby *depotentiating* those synapses (Okuno et al., 2012; **Figure 1**). Further, Arc has been shown to be critical to the pruning, or elimination, of dendritic spines after learning (Nakayama et al., 2015; **Figure 1**). Intriguingly, it has also recently been found that *Arc* can form virus like structures, which encapsulate RNA (Pastuzyn et al., 2018). Neurons have been shown to be capable of taking up these capsids, along with the mRNA contained within (Pastuzyn et al., 2018). This suggests that *Arc* may, in addition to synaptic weakening, enable communication between neurons and thus, though *Arc* is critical to the maintenance of potentiated synaptic strength after LTP induction, whether it is actually involved in the structural changes at the potentiated synapses is unclear.

One of the most critical and well-studied regulators of gene expression associated with L-LTP and LTM is the constitutively expressed transcription factor (TF) cAMP response element binding protein (CREB) (Bourtchuladze et al., 1994; Guzowski and McGaugh, 1997; **Figure 1**). CREB is phosphorylated (pCREB) immediately upon LTP induction, learning or cell firing and leads to an increase in the expression of IEGs (Bito et al., 1996; Deisseroth et al., 1996; Impey et al., 1998; Davis et al., 2000; Benito and Barco, 2010; Benito et al., 2011; Sajikumar and Korte, 2011; **Figure 1**). It is interesting to note that CREB has also been shown to drive increases in intrinsic excitability (Lopez de Armentia et al., 2007; Zhou et al., 2009). Conversely, inhibition of CREB decreases excitability and the induction of L-LTP (Jancic et al., 2009). Learning alone has also been shown to increase excitability (Moyer et al., 1996; McKay et al., 2009) which serves as a means of linking similar learning experiences, and their underlying engrams, which occur within close temporal proximity (< 5 h) (Cai et al., 2016). Indeed, overexpressing CREB has been shown to increase the likelihood of affected neurons being incorporated into an engram (Han et al., 2007; Zhou et al., 2009). Further, some genes regulated by the induction of LTP3 *in vivo*, such as *KCNC2, KCNMA1*, and *CACNG8* encode proteins that regulate excitability (Ryan et al., 2011).

The above evidence indicates that genes that are upregulated by the induction of L-LTP lead to structural and functional changes at both potentiated synapses (via STC), as well as weakened synapses (via inverse or cross-tagging) and may play a role in altering intrinsic excitability. Further, as cell activity alone can drive IEG expression, and LTP3 specifically involves Ca^2+^ influx at the soma, the IEG response may also allow for generalized, cell-wide modifications (**Figure 1**). As a given neuronal ensemble will undergo periods of reactivation and subsequent plasticity, this seemingly broad, non-specific response questions whether the connectivity of an established engram is vulnerable to long-term alteration during periods of heightened gene transcription.

### IEGs Are Critical but Insufficient to Maintain L-LTP

The rapid induction of IEG transcription factor expression has long been associated with persistent LTP. Indeed, when persistent LTP is induced *in vivo* in the dentate gyrus (Abraham et al., 1993), there seems to be a clear correlation between stimulus intensity and IEG expression as well as between stimulus intensity and LTP persistence. There is not, however, a clear relationship between IEG expression and LTP persistence. In response to high frequency electrical stimulation, the expression of IEGs has been shown to increase, with the level of expression generally corresponding to the number of stimulus trains given. Little change is seen after 10 or 20 brief trains, but with 30 trains and above there is a significant increase in expression of transcription factors, *zif/268* (*egr1*), *c-jun and junB*. This coincides with a switch from LTP1 and LTP2 to LTP3 induction (Abraham et al., 1993). However, though 10 trains are insufficient to induce LTP3, 50% of the tested samples did show some increase in the expression of the IEG *Zif/268* (Abraham et al., 1993). After 50 trains, all hippocampi showed increased expression of *Zif/268* even though only 73% showed LTP3 (Abraham et al., 1993). Further, the IEG *c-fos* has also been associated with LTP3 induction but its expression is not reliably altered by all induction paradigms that do induce LTP3 (Dragunow et al., 1989). Thus, although changes in IEG expression are indicative of the induction of persistent plasticity, we speculate that IEGs such as *Zif/268* are part of the plasticity transcriptome which alters connectivity and excitability but does not regulate the persistence of LTP *per se*. Therefore, the question remains: what regulates the persistence of LTP and memory? To answer this question it is fundamental to consider the stage of LTM being investigated.

## Making L-LTP and Memories Persist

The consolidation of LTP and LTM takes considerably longer than the few hours generally studied *in vitro* and *in vivo* (Dudai and Eisenberg, 2004). Numerous experiments have shown LTM to be dependent upon additional transcription- and translation-dependent stages occurring at 12–18 h and 24 h after learning, respectively (Bekinschtein et al., 2007, 2010; Katche et al., 2010). Importantly, infusion of a transcription inhibitor 24 h after learning had no effect if LTM was tested 2 days later, but LTM was significantly impaired if tested at 7 days (Katche et al., 2010). Thus, fundamental elements of the consolidation process occur at distinct times following the initial stimulation.

### Extended Timeframe of Gene Regulation After L-LTP Induction and Learning

Brain Derived Neurotropic Factor (BDNF) has been identified as a potential regulator of a subsequent wave of transcription and translation, after the initial IEG response to learning (Bekinschtein et al., 2007, 2008). Weak learning, which would not normally lead to LTM, could be transformed to LTM by the application of BDNF 12 h after learning (Bekinschtein et al., 2008). Further, BDNF has been shown to be critical for a second wave of expression of the IEGs *c-Fos, Zif/268* (Bekinschtein et al., 2007) and *Arc* (Nakayama et al., 2015) after learning (**Figure 1**). Interestingly, in the case of *Arc* this second wave of transcription may underlie the selective pruning of small mushroom spines, as described above, which is essential to the activation of the engram upon longer term (7 days) but not shorter term (2 days) recall (Nakayama et al., 2015). As discussed above, the ARC-CaMKIIβ interaction can decrease synaptic AMPAR and thus synaptic drive, and CaMKIIβ has also been shown to be critical to long term (10 days) but not short term (1 day) recall (Cho et al., 2007).

### Ongoing Gene Expression After LTP3 and Learning – A Maintenance Transcriptome?

Many of the IEGs that were first identified as regulated after L-LTP induction are themselves inducible TFs (Sheng et al., 1991) and sequential activation of TFs after LTP3 induction *in vivo* has been identified (Williams et al., 2000). This alone suggests that the L-LTP associated transcriptional response is not temporally confined to the proximity of the stimulus. Indeed, extending these early studies, recent transcriptome-wide approaches have confirmed on-going, complex and dynamic changes in gene expression over at least 24 h post-LTP3 induction *in vivo* in the dentate gyrus (Ryan et al., 2012). Furthermore, as described above, subsequent waves of gene expression and protein synthesis have been identified 12 and 24 h after learning, both of which are critical to LTM (Bekinschtein et al., 2008, 2010, Katche et al., 2010, 2012) and, a recent study has shown that sustained neuronal activity, or sustained sensory input, induces waves of different gene expression that are strikingly similar to those which are seen after LTP induction or learning (Tyssowski et al., 2018). However, due to limited information about ongoing gene expression after learning, we focus here on LTP.

Using network analysis of the genes regulated over time after LTP3 induction *in vivo* (Ryan et al., 2012) our group has tried to understand the relationship of altered gene expression to the persistence of LTP. Such analyses aim to derive the role of co-operatively acting groups of genes and identify center hub molecules proposed to play key roles within a given network and we have shown that these hubs are critical to the stability of these gene networks (Nido et al., 2015).

Analysis of the networks regulated rapidly (20 min) post-LTP3 induction suggest that these genes are heavily involved in gene transcription, cell growth and development (Ryan et al., 2011, 2012), matching the restructuring of connectivity and ongoing regulation of gene expression described above. By 5 h post-LTP induction the main function of the genes regulated were calcium dynamics, G-protein signaling and negative regulation of gene expression, functions that regulate the induction of LTP and thus, would affect the induction of subsequent plasticity (Ryan et al., 2012). However, by 24 h the genes regulated were found to be involved in the *inhibition* of protein synthesis and epigenetic *negative* regulation of gene expression. Indeed, in contrast to the earlier times, a general downregulation of gene expression was seen at 24 h (Ryan et al., 2012). To further explore this novel observation, we tested the relevance of the LTP-associated gene networks using random Boolean network simulations. We found that the stability of the early network was crucially dependent on the presence of the key hub molecule, *egr-2* (Nido et al., 2012) and that the 24 network was markedly more stable that the early networks. Further, we also found that the architecture exhibited by a control and the 24 h LTP co-expression networks fit well to a scale-free distribution, known to be robust against perturbations (Nido et al., 2015), remarkably mirroring the stability of LTP and memory. Together these observations support the novel hypotheses that the waves of gene expression observed following LTP3 play divergent roles over time and that there may be a shift in the threshold for plasticity related gene expression over time after LTP3 induction, underpinned by epigenetic modifications.

## A Role for the Inhibition of Plasticity in the Maintenance of LTP and Memory

An engram is initially labile and susceptible to disruption but is then consolidated and made resistant (Dudai, 1996; Frankland and Bontempi, 2005; Medina et al., 2008; Alberini and Kandel, 2015). The extinction of a previously learned fear behavior is a commonly used tool that can probe the structural stability, and integrity, of an engram. However, much debated has focused on whether the original fear engram is altered during fear extinction, or if the fear extinction training creates an entirely new engram. A compelling study has recently shown a positive correlation between the number of cells activated during fear extinction that were part of the original fear memory engram and the efficiency of the new fear extinction training (Khalaf et al., 2018). This suggests, at least in part, that the integrity of the original fear engram was detrimentally effect by the recruitment of those cells to the new memory, i.e., the fear memory was diminished or lost (Khalaf et al., 2018). Thus, some break that inhibits plasticity within a neuron incorporated into an engram would be advantageous to the protection of said engram. Indeed, in some conditions once LTP has been induced or learning has occurred, further plasticity, even at synapses that were not potentiated, is blocked or occluded (Abraham et al., 2001; Whitlock et al., 2006; Hulme et al., 2012; Nabavi et al., 2014). In the motor cortex this occlusion can be maintained for at least 23 days (Rioult-Pedotti et al., 2007). This increase in the threshold for plasticity may allow the engram to persist over that time by blocking other inputs from making competing alterations. Indeed, sufficiently strong competing inputs have been shown to detrimentally affect the persistence of LTP. Inducing LTP3 at one set of synapses on the DG granule cells can cause a previously established LTP3 at a different set of synapses to decay rapidly (Abraham et al., 2006). These results suggest that subsequent LTP induction may be detrimental to previously established LTP.

### Reactivating an Engram Induces a Labile State That Can Disrupt Its Connectivity

Reactivation of the engram can make it labile again and allow it to be updated with new information (Ramirez et al., 2013, 2015; Khalaf et al., 2018). Over time, after an engram has been updated, it can be reconsolidated, a process which is dependent upon very similar mechanisms to learning and consolidation, including NMDAR receptor activation, protein synthesis and gene expression, and seemingly works by STC mechanisms (Przybyslawski and Sara, 1997; Alberini, 1999; Nader et al., 2000; Nader and Hardt, 2009; Cassini et al., 2013). Surprisingly though, with regards to LTP, reactivating a pathway 24 h after LTP had been induced in that pathway, using the same stimulation protocol, the expression of IEGs is significantly less than that after the initial LTP induction (Abraham et al., 1995). This suggests that IEG expression is in some way dampened by prior activity. Support for this comes from the visual cortex, where plasticity mechanisms are severely attenuated after what is described as the “critical period” is closed (Hubener and Bonhoeffer, 2014). Dampened expression of the IEG Arc is fundamental to the reduced plasticity and it can in fact be rescued by simply increasing levels of Arc in the region (Jenks et al., 2017). In addition, an epigenetic break on transcription (see Section 5) has been shown to limit the lability of a stable memory and, if alleviated, long-term plastic changes can be made to an established engram (Gräff et al., 2014). Together, these studies imply that different activation thresholds for gene expression exist and accordingly, reactivation of an engram may induce a different gene expression response than that induced during the initial experience (Harrow et al., 2012). Further, it is possible then that decreasing the efficiency of the signaling pathways or engaging an epigenetic mechanism to dampen gene expression could achieve this transcription response.

### Genes Which Negatively Regulate Plasticity Are Themselves Upregulated 24 h Post-LTP: A Proposed Maintenance Transcriptome

**FIGURE 2:**
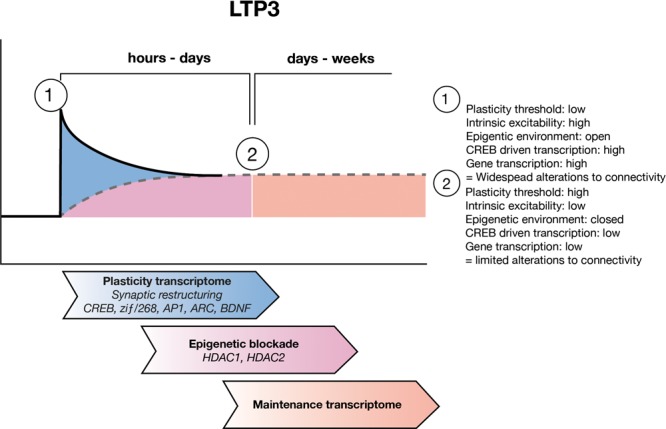
LTP maintenance hypothesis. Upon LTP induction, the threshold for plastic adaptations throughout the cell is low and as such CREB driven gene expression allows for widespread structural and functional modifications to synapses and to the intrinsic excitability of the cell. Over the subsequent hours to days, ongoing waves of gene expression allow for refining of the engram circuitry, through pruning of unwanted inputs. During this time the maintenance transcriptome develops which includes epigenetic negative regulators of gene expression such as HDAC1 and HDAC2 as well as other negative regulators of plasticity. Eventually the maintenance transcriptome sets a new, higher threshold for plasticity which allows for the connectivity of the engram to be preserved.

The TF, nuclear factor kappa-light-chain-enhancer of activated B cells (NF-κB), is the central hub of one of the most important networks of genes found to be regulated 24 h post-LTP3 (Ryan et al., 2012). NF-κB is activated after LTP3 induction (Freudenthal et al., 2004), is critical to memory reconsolidation, in particular via epigenetic regulation of gene expression (Lubin and Sweatt, 2007), and the maintenance of memory (Levenson et al., 2004a ; Dash et al., 2005; Freudenthal et al., 2005). The network, which largely contained upregulated genes, includes the *Tnf receptor, MAPK13* and *MAPKAPK3* (Ryan et al., 2012). These genes are involved in the TNFα - p38 MAPK pathway, which has previously been implicated in negative modulation of LTP induction (Gisabella et al., 2003; Butler et al., 2004; Izumi et al., 2008; **Figure 2**).

A second important network of genes regulated 24 h post-LTP3 induction, involved a considerable number of downregulated genes, with a central hub of histone deacetylase 2 (HDAC2) which is itself upregulated (Ryan et al., 2012). Interestingly, histone deacetylase 1 (HDAC1) was also part of this network but was instead downregulated, though it was upregulated and central to the strongest network of genes regulated 5 h post-LTP induction (Ryan et al., 2012). Histone acetylation is an important epigenetic mechanism that controls gene expression and is regulated via histone acetyltransferases (HATs) and deacetylation via histone deacetylases (HDACs), which have been strongly associated with memory, LTP and memory-related diseases such as Alzheimer’s disease (Levenson et al., 2004b; Guan et al., 2009; Gräff et al., 2012). HATs acetylate lysine residues on histone tails, creating an environment that is permissive to gene transcription by allowing TFs access to their target genes (Gräff et al., 2011; Stilling and Fischer, 2011). HDACs remove acetyl groups from lysine residues on histone tails, thereby decreasing the permissiveness to gene transcription and are thus believed to be negative regulators of gene expression, L-LTP induction and learning (Gräff et al., 2011; Stilling and Fischer, 2011). Due to the central, high-level control of gene expression which epigenetic mechanisms employ, and the fact that we found HDAC1 and 2 to be regulated by the induction of LTP3, we propose that the change in HDAC expression we identified may be indicative of a metaplastic rise in the threshold for plasticity related gene expression. In conjunction with signaling pathways such as the p38 – MAPK discussed above, this general inhibition of plasticity may protect the structure and function of the cell involved in the engram (**Figure 2**). It is important to note that while we have focused this hypothesis on HDAC1 and 2 due to our findings, numerous other HDAC family members have been found to be important for learning, memory and synaptic plasticity (Mahgoub and Monteggia, 2014; Penney and Tsai, 2014).

## A Role for Histone Acetylation As An Epigenetic Metaplasticity Mechanism To Regulate Engram Maintenance

### A Permissive Epigenetic State Coincides With the Induction of L-LTP and Learning

The vital role of gene transcription for the persistence of LTP and memory leads to the hypothesis that an epigenetic environment that is permissive to gene transcription would coincide with periods of heightened gene transcription. Upon learning, histone acetylation is transiently increased (Alarcón et al., 2004; Wood et al., 2005; Chwang et al., 2007; Vecsey et al., 2007; Miller et al., 2008; Gräff and Tsai, 2013) through an NMDAR and ERK-dependent pathway (Levenson et al., 2004b). Further, upon repeated learning experiences, acetylation is also increase within an hour (Bousiges et al., 2010). Aged animals with LTM impairments do not display an increase in acH4K12 after fear conditioning, which occurs transiently in control animals (Peleg et al., 2010). Though only correlational, this suggests that at least a brief increase in acetylation may be critical to the formation of LTM. Additionally, evaluation of the epigenetic environment after neuronal activity alone has shown that genes that respond rapidly to activity have a more open, permissive chromatin state and have more pre-engaged transcriptional machinery than those that respond more slowly or only to long-durations of activity (Tyssowski et al., 2018). Interestingly, this group of rapidly responsive genes also depend on the activation of the MAPK/ERK pathway (Tyssowski et al., 2018).

### Creating a Permissive Epigenetic Environment Promotes Plasticity

Artificially increasing histone acetylation has been shown to promote L-LTP. Incubation of hippocampal slices with the HDACi trichostatin A (TSA) or sodium butyrate (NaBut) increases acetylation and facilitates both the induction and maintenance of LTP (over 3 h) in a transcription-dependent manner (Levenson et al., 2004b). However, the enhanced magnitude of LTP upon induction suggests that HDAC inhibition itself must drive transcription under basal conditions, i.e., prior to, rather than only in response to, LTP induction. Further, application of TSA to slice preparations, prior to and throughout recordings, has been shown to promote the induction of L-LTP from stimuli that would normally only induce E-LTP (Vecsey et al., 2007). Again, this was found to be critically dependent upon transcription, in particular CREB driven gene expression (Vecsey et al., 2007).

Enhanced learning is also evident after increasing acetylation. Rats exposed to the HDACis vorinostat or TSA show enhanced fear conditioning and object recognition when tested 24 h post-learning (Vecsey et al., 2007; Stefanko et al., 2009; Fujita et al., 2012). Further, modified animals in which HDAC2 is knocked out (HDAC2KO) display enhanced fear conditioning and spatial learning at 24 h, whereas in animals that over-express HDAC2 (HDAC2OE) animals display the opposite (Guan et al., 2009). Indeed, vorinostat is also known to enhance fear extinction 24 h after injection (Fujita et al., 2012) and both TSA and vorinostat seemingly lead to this enhancement via increased acetylation and pCREB driven gene expression (Vecsey et al., 2007; Fujita et al., 2012). However, although HDAC2KO animals have enhanced contextual conditioning at 24 h post-learning, this is not retained at later time points (Morris et al., 2013). In fact, *cued* fear conditioning was not enhanced at 24 h and was actually decreased 48 and 72 h after learning. This effect has been attributed to enhanced fear extinction (Morris et al., 2013) though it could also be argued that LTM was inhibited. Finally, increasing acetylation simply by exposing animals to an enriched environment can restore learning capabilities in the ck-p25 transgenic mouse model of neurodegenerative diseases (Fischer et al., 2007).

### Decreased Histone Acetylation Restricts Plasticity

In accordance with enhanced LTP in a permissive epigenetic environment, a restricted epigenetic environment is detrimental to L-LTP. The CREB co-activator CBP functions as a HAT and therefore increases acetylation. In hippocampal slices from heterozygous CPB knockout mice (cbp^+/-^), E-LTP was not affected but L-LTP was significantly diminished (Alarcón et al., 2004). This reduction was associated with a reduced level of acetylation. By reducing HDAC activity using the HDACi Suberoylanilide Hydroxamic Acid (SAHA) prior to LTP induction, presumably thereby restoring balance between HAT and HDAC activity, the magnitude of L-LTP was restored to control levels (Alarcón et al., 2004). Together, these data suggest that at the time of learning or LTP induction, the transcription of genes necessary for persistent LTP or memory is regulated by an increase in histone acetylation. This epigenetic state, which is permissive of gene transcription and formation of LTM, may also exert a paradoxical side effect where the previous connectivity of a given neuron is made vulnerable to disruption at this early stage.

The cbp^+/-^ mice (Alarcón et al., 2004) as well as mutant mice which lack the HAT ability of CBP (CPB HAT^-^) (Korzus et al., 2004), and mice which lack the CREB isoforms α and Δ (Vecsey et al., 2007), all show impaired LTM formation. Intraventricular infusion of the HDACi SAHA increased acetylation and recovered memory to control levels in the cbp^+/-^ mice (Alarcón et al., 2004). However, the impairment seen in the CREB mutants could not be restored to the same degree (Vecsey et al., 2007). This suggests that, as in LTP experiments, TSA treatment must work via CREB to enhance learning. Thus, the recovered acetylation and memory in cbp^+/-^ animals, in response to the HDACi, seemingly overcame the limited HAT capabilities either by driving CREB dependent transcription and/or by decreasing HDAC activity (Alarcón et al., 2004; Vecsey et al., 2007). Interestingly, the CPB HAT^-^ mice showed significant impairment in recognition and spatial memory tasks. However, this impairment could be rectified by recovery of HAT activity or by more intensive training (Korzus et al., 2004). Similarly, cbp^+/-^ animals that were repeatedly trained in the spatial memory task had no deficits (Alarcón et al., 2004). This suggests that the permissive, open state of the chromatin can be brought about via a number of mechanisms regulating the balance of HAT and HDAC activity. However, it is achieved, increased acetylation is needed to open the chromatin nearby plasticity related genes.

### HDAC2 Negatively Regulates the Capacity for Structural Plasticity of Synapses

In support of the hypothesis that the plasticity transcriptome underpins synaptic restructuring, knock-out of the *Hdac2* gene (HDAC2KO) causes long-term structural modifications to synapses. Slices from HDAC2KO mice display decreased synaptic transmission (Morris et al., 2013), while in another set of experiments using HDAC2KO mice, despite an overall increase in spine density, a decreased number of mushroom spines was found (Guan et al., 2009). On the other hand, spine density was decreased in *Hdac2* over-expressing animals (HDAC2OE) (Guan et al., 2009) and in a disease model which leads to *Hdac2* over-expression (Gräff et al., 2012). Thin spines are believed to potentiate into mushroom spines upon LTP induction, a process hypothesized to be a structural trace of memory formation (Bourne and Harris, 2007). Although it has not been conclusively shown, the combination of decreased synaptic transmission and fewer mushroom spines in HDAC2KO mice, despite greater overall spine density, suggests that the existing spines contain silent or non-potentiated synapses with fewer AMPARs (Matsuzaki et al., 2004). Increasing the number of silent spines has previously been shown to enhance LTP induction (Arendt et al., 2013), presumably because he number of spines at which LTP can be induced is increased. Thus, these results would predict that HDAC2KO or KD animals should have enhanced L-LTP induction. Indeed this is the case, as L-LTP can be induced with mild stimulation, which would normally only induce E-LTP, in hippocampal slices from HDAC2KO mice (Guan et al., 2009; Morris et al., 2013) and L-LTP could not be induced in HDAC2OE animals (Guan et al., 2009) or in a disease model which leads to HDAC2 over-expression (Gräff et al., 2012). Together, these results suggest that HDAC2 negatively regulates the expression of genes associated with spine growth and synapse formation, and this may lead to enhanced learning. Intriguingly, the lack of mushroom spines in the HDAC2KO mice suggests that this may be at the expense of maintaining LTP and LTM.

### Evidence of HDAC1 and 2 Regulation of the Plasticity Transcriptome

With the promotion of structural plasticity at spines and synapses, at the expense of maintenance, it would be expected that HDAC2 would negatively regulate genes related to this process. The level of acetylation and HDAC2 present at the promoter region of a number of important plasticity and neuronal activity related genes are inversely related in HDAC2OE and KO animals (Guan et al., 2009). Most importantly, genes required for structural rearrangement during learning and in synapse formation (such as *CaMKIIa and PKMζ)* are directly regulated by HDAC2. Concomitant mRNA expression of these genes has also been identified (Guan et al., 2009). Further, in an Alzheimer’s disease model which had increased HDAC2 expression, genes involved in structural rearrangement of synapses such as *Arc* and *Homer1* were suppressed, as were genes related to synapse formation such as *Glur1, Glur2, Nr2a, Nr2b* and *Syp* (Gräff et al., 2012). Together, these observations support the learning and LTP results suggesting HDAC2’s negative regulation of learning and plasticity, rather than maintenance *per se*. Surprisingly, HDAC2OE animals also showed *increased* acetylation at the promoter region of some plasticity related genes (Guan et al., 2009). While regulation of HDAC2 levels did, in most cases, have expected results on acetylation, there were significant exceptions to this rule (Guan et al., 2009). However, a genome-wide analysis of HDAC2 expression has not been performed and as such, the specificity of HDAC2 regulation of plasticity related genes cannot be concluded at this time.

Histone deacetylase 1 (HDAC1) overexpression has been shown to lead to either no change in the acetylation at the promoter region of most genes investigated, or lead to an increase in acetylation of some plasticity related genes (Guan et al., 2009). Confusion around the implications of this is exacerbated by the finding that the loss of HDAC1 activity through a neurodegenerative disease model and through knockdown by siRNA resulted in neuronal cell death with an associated increase in expression of genes related to cell cycle (Kim et al., 2008). These effects could be ameliorated by overexpression of HDAC1 (Kim et al., 2008). Further, HDAC1 has been shown to gradually accumulate at the promoter region of *c-Fos*, over 5 days of fear extinction training, which parallels a gradual decrease in *c-Fos* expression over the same time course (Bahari-Javan et al., 2012). Although these results highlight a mostly tight negative regulation of genes involved in plasticity and learning by HDAC2, there are clear exceptions to this in mRNA expression and acetylation and cast some doubt over the dogma that HDACs negatively regulate acetylation, gene expression and LTM.

### Ongoing Epigenetic Regulation

The evidence presented so far supports the notion that HDAC2 in particular regulates the expression of genes involved in structural rearrangements of synapses in the early time period after learning or the induction of L-LTP. However, as described above, there is evidence that dynamic gene expression occurs for at least 24 h, such as via BDNF driven transcription (Bekinschtein et al., 2008). Therefore, dynamic *regulation* of gene expression over this time should also occur. Recently it has been shown that *Arc* gene expression is driven by different response elements within the promoter region of the gene (Fukuchi et al., 2015). Specifically, it was found that the synaptic activity-response element (SARE), located -7 kbp upstream of the *Arc* transcription start site (TSS), was responsive to NMDA, BDNF and FGF2 but that a proximal promoter region, -1679 from the TSS was only responsive to BDNF and FGF2 (Fukuchi et al., 2015). Upon induction of LTP, presumably through NMDAR driven transcription and thus derived from activation of the more distal response element, *Arc* has been shown to move out of the nucleus toward active dendrites (Steward et al., 1998, 2014). However, in neuronal cell culture after NMDAR activation, ARC has been shown to localize in the nucleus and interact with Tip60, a HAT, leading to increased acH4K12 (Wee et al., 2014). The second wave of *Arc* expression, described above as critical to LTM, is driven by BDNF (Nakayama et al., 2015). The proximal promoter region of *Arc*, regulated by BDNF, is under HDAC1 control (Fukuchi et al., 2015). Interestingly, it was found that the HDACi TSA could only enhance the transcription of *Arc* by this proximal promoter region (Fukuchi et al., 2015). In striking contrast, TSA actually inhibited NMDA-driven expression (Fukuchi et al., 2015). This suggests that HDACi driven gene expression is changing the plasticity-induced transcriptome and, therefore, the tight control of transcription may be lost. As *Arc* can be regulated by HDAC2 (Gräff et al., 2012) and HDAC1 (Guan et al., 2009; Fukuchi et al., 2015), there may be waves of *Arc* expression, potentially driven by different promoters and regulated by different epigenetic mechanisms. If the expression of one gene is dynamically regulated by different signaling mechanisms and under the control of different HDACs over time, there is potential for other genes to have similar capacities and thus tight temporal regulation of the epigenome may be extremely important.

### HDAC Activity as a Metaplastic Protector of Engrams

The critical question for our hypothesis is whether enhancing a permissive chromatin state destabilizes previously established engrams. Ocular dominance columns of the visual cortex are well-characterized examples of highly stable neuronal networks. After a critical period of plasticity during early post-natal days, the visual cortex becomes rigid and signaling pathways which would normally induce the expression of plasticity genes are unable to do so (Putignano et al., 2007). Evidence suggests that though the intracellular pathways involved are still fully functional, the gene expression response is suppressed, and this suppression is regulated by either increased HDAC activity, or by decreased HAT activity (Putignano et al., 2007; Baroncelli et al., 2016). Increasing histone acetylation with an HDACi can lead to the destabilization of the neuronal networks forming the ocular dominance columns and allow for restructuring and updating of the circuitry (Putignano et al., 2007; Lennartsson et al., 2015; Baroncelli et al., 2016).

Updating of a conditioned fear response, much like restructuring of ocular dominance columns, similarly tests the strength and maintenance of the original connectivity related to a fear response. Indeed, a fear memory, 24 h post-learning, is labile and can be readily and persistently updated with fear extinction training (Gräff et al., 2014). In conjunction with this, acetylation of H3K9/K14 is significantly increased 1 h after recall of this recently formed memory (Gräff et al., 2014). However, with a consolidated, 30 day old fear memory, there is no such increase in acetylation upon recall, and the memory cannot be persistently altered by the same extinction protocol (Gräff et al., 2014). This suggests negative regulation of acetylation coincides with negative regulation of genes involved in the structuring of connectivity, described above. Indeed, inhibiting HDAC2 before the recall of the 30 day old memory allows for IEGs such as *Arc* and *c-Fos* to be expressed in response to recall, and for a persistent alteration to the memory to be made (Gräff et al., 2014).

Histone deacetylase 1 (HDAC1) has been shown to positively regulate fear extinction (Bahari-Javan et al., 2012), seemingly opposite to the idea that negative regulators of gene expression negatively regulate LTM. Acute overexpression of HDAC1 enhanced fear extinction, however, it had no effect on working memory, novel object recognition or contextual fear conditioning (Bahari-Javan et al., 2012). A gradual decrease in *c-Fos* expression after each fear extinction trial is shown to correspond to an increase in HDAC1 and a decrease in acH3K9 at the promoter region of the *c-Fos* gene at the same time points (Bahari-Javan et al., 2012). Thus, HDAC1 appears to negatively regulate the expression of *c-Fos*, altering the reconsolidation of the engram to decrease the fear response. These examples suggest increased regulation of histone acetylation does play a role in maintaining consolidated memories over time by raising the threshold needed to be reached for plasticity related gene expression to occur.

Evidence supports another epigenetic transcriptional repressor as a maintenance mechanism LTM (Miller et al., 2010). Long-term changes in methylation of GC-rich CpG islands in the dorsomedial prefrontal cortex has been shown to be critical to the maintenance of LTM (Miller et al., 2010). These changes were distinct from epigenetic changes in the hippocampus during the initial learning period and suggested to perhaps underlie state changes to the threshold for plasticity or to the ongoing synthesis of proteins that support potentiated synapses (Miller et al., 2010). Further, Webb et al., 2017 propose that histone methylation and DNA hydroxymethylation regulate the expression of specific genes during retrieval of recent vs. older memories in a region specific manners (Webb et al., 2017). There is also evidence for early changes in methylation state also correlate with expression of plasticity related genes after LTP induction *in vivo* (though in anesthetized animals) (Maag et al., 2017). Much like acetylation, how methylation plays a role in learning versus memory is still under discussion (Oliveira, 2016).

While most of the above information would suggest that HDACs negatively regulate gene expression, it has been shown that HDAC1 and 2 are recruited to active genes (Wang et al., 2009). Further, the HDACi TSA, which has been used in a number of experiments, has been shown to largely affect regions of the genome which were found to be enriched in acH4K9/K14 and trimethylated H3K4 (H3K4me3) at basal levels and indeed these marks were a prerequisite for H4 hyperacetylation in response to TSA (Lopez-Atalaya et al., 2013). This indicates that the effect of TSA is restricted to regions of the genome that are already somewhat active. Together, these results suggest that HATs and HDACs must be working at the same promoter regions to regulate the balance of acetylation (Wang et al., 2009; Lopez-Atalaya et al., 2013). Finally, it is also important to remember that HDAC1 and 2, like other HDACs, have non-histone targets such as p53, E2F1, GATA4 and NF-*κ*B, in particular the p65 subunit (Kelly and Cowley, 2013).

## Summary

Here, we have built on the evidence that L-LTP and LTM requires not only potentiation of synapses but also depotentiation or depression of neighboring synapses, and alterations to intrinsic excitability. We suggest that all of these changes occur together as a concerted response and are the result of a specific plasticity transcriptome, which produces gene products that make changes to the structure and function of the cell (**Figure 2**). However, we propose that the plasticity transcriptome does not underlie maintenance of structural changes involved in engram formation and thus, LTM. We hypothesize that there is also a maintenance transcriptome, particularly controlled by the action of HDAC1 and HDAC2 activity but other epigenetic regulators may also be involved, which acts to raise the threshold at which future activity can again induce the expression of the plasticity transcriptome, helping to ensure that the integrity of the engram is preserved (**Figure 2**).

Dynamic gene expression profiles play specific roles at different stages during the consolidation of L-LTP, LTM and the engram structure. In particular, IEGs related to the restructuring of synaptic connections upon learning or LTP induction seem to be strongly regulated by HDAC2. However, the profiles identified at later time points, particularly during the stabilization of the engram, have been less well studied. Three critical components must be investigated more thoroughly to test our hypotheses. First, the epigenetic state of cells involved in a given engram, at various time periods after learning needs to be established to determine if there is in fact an epigenetic identity of an engram. Secondly, subtype-specific HDACis must be identified, rather than relying on broad-spectrum HDACis that do not differentiate between HDAC1 and 2. Thirdly, the effects of HDAC inhibition on LTM and the persistence of LTP need to be assessed at stages later than 24 h post-learning. Together, these three components will then allow for testing of the stability of an engram in the face of new learning, and whether there is in fact an epigenetic metaplasticity mechanism involved in the maintenance of memory.

The determination to find memory-enhancing drugs which may aid in the treatment of neurodegenerative diseases in particular, such as HDACis, needs to be met with a thorough understanding of what underlies the long-term maintenance of memories in the first place. Systemic application of broad-acting inhibitors has the potential to create a highly plastic learning environment but may come at the expense of a stable, long-term storage environment. Thus, in a disease state in which reduced plasticity is a symptom, is recovering that plasticity always the best option for the health and survival of the cell in question? Regardless of the answer, understanding the learning and maintenance processes of memory, and why these may go awry in disease states, is critical for the specific targeting of treatments as well as for the basic understanding of brain function.

## Author Contributions

All authors listed have made a substantial, direct and intellectual contribution to the work, and approved it for publication.

## Conflict of Interest Statement

The authors declare that the research was conducted in the absence of any commercial or financial relationships that could be construed as a potential conflict of interest.
